# A case of bacteremia and pneumonia caused by *Streptococcus equi* subspecies *equi* infection in a 70-year-old female following horse exposure in rural Wyoming

**DOI:** 10.1186/s12941-023-00602-1

**Published:** 2023-08-02

**Authors:** Tristan Bohlman, Heith Waddell, Brant Schumaker

**Affiliations:** 1 University of Washington School of Medicine, Seattle, Washington, USA; 2 University of Washington School of Medicine, Seattle, United States; 3 Wyoming WWAMI University of Washington School of Medicine, University of Wyoming, Laramie, Wyoming, USA

**Keywords:** Zoonoses, *Streptococcus equi*, Bacteremia, Equine

## Abstract

**Background:**

The occurrence of zoonotic infections following an animal exposure continues to be an important consideration for all patients, especially those within agricultural communities. *Streptococcus equi* subspecies *equi* (*S. equi* subsp. *equi*) is a bacteria known to cause a common infection called ‘Strangles’ in horses. This article highlights a new case of pneumonia and bacteremia in a patient caused by *S. equi* subsp. *equi* following strangles exposure in a horse. Rarely has there been reported horse to human transmission of subsp. *equi*.

**Case Presentation:**

A 70-year-old woman attended a rural emergency department with complaints of dry heaving, fever, chills, shakes, and nausea and presented with a cough. She had undergone a screening colonoscopy two days prior with no other significant medical history. The patient had computed tomography (CT) evidence of a pneumonia and positive blood cultures growing *S. equi* subsp. *equi* consistent with bacteremia. The patient later disclosed the recent passing of her horse following its sudden illness six days prior to her emergency department presentation. She had cuddled and kissed the horse prior to its death. The patient was treated with IV lactated ringers during the initial evaluation and admission and also received IV piperacillin-tazobactam 4.5 g every eight hours intravenously during her hospital stay. She was transitioned to an oral antibiotic on discharge. Subsequent blood cultures drawn the day after discharge were negative for *S. equi* subsp. *equi*, indicating successful treatment of her bacteremia.

**Conclusions:**

This report discusses an atypical presentation of *S. equi* subsp. *equi* infection in an otherwise healthy individual, manifesting as early sepsis, pneumonia, and bacteremia. The patient likely developed this infection following direct contact exposure to her horse who had died from presumed strangles a few days prior to her symptom onset. This case highlights the importance of investigating potential exposures to *S. equi* subsp. *equi* in rural areas, areas where farming and ranching are prevalent, particularly among individuals working with horses. It is especially important to acknowledge high risk populations such as immunocompromised individuals with signs and symptoms of meningitis or bacteremia.

## Background

Zoonotic disease transmission from animals to humans has been described in medicine for centuries. A recent case demonstrating transmission of *Streptococcus equi* subspecies *equi* (*S. equi* subsp. *equi*) from a horse to a human contact is exceedingly rare [1]. *S. equi* is a Lancefield group C β-hemolytic streptococci; a Gram positive bacteria that often causes mucosal diseases in various mammalian species [2]. *S. equi* has two subspecies which cause zoonoses, including subspecies *zooepidemicus* and *equi* [2]. Of these, *S. equi* subsp. *equi* is the pathogen known for causing the disease ‘strangles’ in horses [2]. Importantly, bacteria belonging to serogroup C, such as *S. equi*, are the cause of 0.25 to 7.2% of all streptococcal infections in horses [2]. Strangles is of high importance in horses due to its potential clinical severity and high transmissibility among animal contacts. In fact, it is one of the most commonly encountered equine clinical problems [2]. Transmission to humans may be a result of sharing mucous secretions with an infected animal, or any other method of contact with secretions that expose the individual to large amounts of the pathogen [2]. In a review of the literature conducted in 2010, 31% of the reported cases of serogroup C infection to humans arose from equine exposure, while 19% followed ingestion of dairy products [1]. Another literature review in 1991 evaluated group C streptococci infections in humans and found that the most common clinical findings were endocarditis (27.3%), primary bacteremia (22.7%), and meningitis (10.2%) [3]. While these findings are insightful, only two of the eighty-eight cases in this review were due to *S. equi* subsp. *equi* and the majority were unable to be identified, making it difficult to use these findings in guiding clinical evaluation and practice.

*S. equi* infections in humans is a fairly rare phenomenon [1, 4]. In recent literature searches at the time of submission, only one case of horse to human infection by *S. equi* subsp. *equi* has been reported in the United States [5]. This case report describes a patient with early sepsis, bacteremia, and pneumonia following an exposure to secretions of a horse with probable active strangles. Understanding potential clinical presentations of humans infected with this pathogen is crucial to enact timely diagnosis and treatment. This may be seen particularly in areas where ranching, farming, or other animal-related occupations and contacts are prevalent.

## Case presentation

A 70-year-old female presented to a rural emergency department with complaints of dry heaving, fevers, chills, shakes, and nausea. Of note, this was two days following a colonoscopy procedure for routine screening. The dry heaving and nausea began one day postoperatively. Despite an uneventful colonoscopy, per the surgeon, the patient and spouse expressed concern about a procedural complication, including a possible perforation. The patient denied any abdominal pain. She also denied any chest pain or shortness of breath, but did have a significant cough at the time of presentation to the emergency department. Regarding her medical history, the patient had no medical conditions or recent illnesses that would compromise her immune system. She denied taking any prescription medications.

Vital signs on admission demonstrated a blood pressure of 164/98 mmHg, heart rate 110 beats/min, respiratory rate of 20 breaths/min, saturations of 94% on room air and an oral temperature of 39.7^o^C. The physical examination noted a tired looking individual with a normal exam except for crackles auscultated in the right base and mid lung. No murmurs were auscultated on exam. Labs and imaging were initiated during the evaluation in the emergency department. The laboratory panels revealed normal electrolyte values with a leukocytosis of 11.5 × 10^3^/µL and neutrophilia of 88%. Venous blood gases revealed a respiratory alkalosis (pH − 7.533; PO2–47.5 mmHg; and PC02–19.4 mmHg). Lactic acid was normal at 1.20 mmol/L, and hemoglobin was normal at 14.0 g/dL. Her urinalysis revealed ketones and leukocyte esterase. This was later found to contain moderate squamous epithelial cells which was not satisfactory for culture. The patient did not note urinary symptoms, another sample was not obtained. An electrocardiogram performed in the emergency department showed sinus tachycardia. Blood cultures were also taken and sent for culture to further identify potential contributors of early sepsis or bacteremia.

Sepsis was certainly considered due to the patient’s tachycardia, leukocytosis, reported fever and chills, a downward trend in blood pressure from initial intake measurement to 97/60 mmHg and her recent colonoscopy procedure. Diagnostic imaging studies were performed, including chest radiographs and abdominal/pelvic computed tomography (CT). Chest radiograph was reported as normal. CT of the abdomen/pelvis was performed due to the recent procedure. It demonstrated [1] no acute intra-abdominal abnormality and [2] patchy ground glass opacities in the right middle lobe that were nonspecific and could be indicative of an atypical infection or atelectasis. Due to her leukocytosis and the nonspecific findings on chest X-ray, it was felt to be most beneficial for the patient to be admitted to the hospital. She was started on piperacillin-tazobactam 4.5 g every eight hours intravenously to address her nonspecific lung findings and potential bacteremia. At this point in time, the most likely diagnoses included early sepsis, a bacteremia, possibly an aspiration pneumonia versus an atypical pneumonia, with no evidence of intra-abdominal pathology.

The day following hospital admission, the patient’s vital signs had returned to normal values with a blood pressure of 119/74 mmHg, a heart rate of 80 bpm, and a temperature of 36.8 °C. She denied chills, shakes, shortness of breath, or chest pain and had no difficulty ambulating. The blood cultures taken the day prior in the emergency department returned positive for *Streptococcus* by polymerase chain reaction (PCR). All culture bottles, confirmed positive by PCR were negative for Group A, Group B, and *S. pneumoniae*. Due to the possibility of the *Streptococcus spp.* being speciated as a pathogen like *S. viridans* or another organism that could cause endocarditis or serious infection, the patient was advised to continue IV antibiotics and remain in the hospital. At this time, she did not have any typical features of endocarditis, such as tachycardia, fever, chills, aching joints, night sweats, or edema. The patient’s white blood cell count had improved to a normal value of 8.4 × 10^3^/µL. Her neutrophilia had also improved overnight, to normal values from 88 to 67.8%.

Two days post-hospital admission, the patient’s leukocytosis (5.7 × 10^3^ µL) and neutrophilia (60.6%) had normalized, and she had no further symptomatology of chills, nausea, or dry heaving throughout her hospital course. Her blood culture isolate previously identified as *Streptococcus* via PCR was subsequently typed as *S. equi* subsp. *equi* by MALDI-TOF mass spectrometry. This result was puzzling, as a bacteremia from *S. equi* is not a common pathogen to cause either a bacteremia or a pneumonia in humans. After this finding, the patient was asked about her exposure to strangles, which is the presentation of a *S. equi* subsp. *equi* infection in horses. During the emergency department visit, the patient had discussed with the physician the recent and unexpected passing of her horse. After receiving the blood culture results, the patient disclosed that, while the horse was passing, she had been kissing and cuddling it. Her husband described initially finding the horse as being lethargic, having had a respiratory change with increased work of breathing, holding its head down and looking sick. The horse expired the next morning with significant mucous and blood from its nostrils and mouth. Her horse passed six days prior to her presentation at the emergency department. Due to the epidemiologic evidence, horse to human transmission of *S. equi* was suspected. Antibiotic susceptibility testing showed that the organism was susceptible to all forms of penicillin. The patient was discharged on amoxicillin (1 gram three times daily for 12 days) and remained asymptomatic. On repeat blood cultures drawn one day following her discharge from the hospital, there was no growth of *S. equi* or other bacteria at 102 h. The patient followed up with the primary care clinic two and a half weeks post discharge and again at seven weeks with the physician who was also the admitting physician for this patient. At that visit the patient felt she was at her baseline.

In this case, the horse presumed to be the source of the patient’s infection was not able to be swabbed for culture, as the horse died six days prior to the patient’s presentation to the emergency department. Thus, a diagnosis of strangles in the horse was not confirmed. However, due to the symptoms of the horse that the patient described, her subsequent close contact with the horse, the patient’s blood cultures, and a conversation with the local veterinarian, it can be reasonably deduced that the horse was the source of the *S. equi* subsp. *equi* infection.

## Discussion and conclusions

Following any procedure, it is important to be aware of potential complications, especially when a patient presents with signs of early sepsis. In the present case, consideration for an intraoperative complication was important, this differential diagnosis was quickly ruled out by CT and abdominal exam. Importantly, there was evidence of pneumonia on thoracic CT and, later, a bacteremia with a rare human pathogen, *S. equi.*

As described previously, *S. equi* has two primary subspecies that cause infection: *zooepidemicus* and *equi* [2]. *S. equi* subsp. *zooepidemicus* is a part of a horse’s natural nasopharyngeal flora [6]. It is also more commonly a disease-causing pathogen in humans with numerous case reports highlighting its prevalence [7–10]. However, *S. equi* subsp. *equi* less commonly causes human infection and is the pathogen responsible for the horse disease ‘strangles’. This group C bacteria most commonly infects the nasal passages, pharynx, and lymph nodes of horses, potentially causing compression of the upper airway due to abscessation of the lymph nodes in progressed disease states [6]. *S. equi* evades the host’s immune system via its M-protein, which shares approximately 80% of its genome with the human pathogen *S. pyogenes*, as well as its hyaluronic acid capsule [6, 11]. The organism efficiently evades the host immune system, resulting in the formation of retropharyngeal lymphadenopathy or abscesses [6]. Many horses will develop a mucopurulent discharge where transmission of the pathogen is very efficient among horses or other mammals in close proximity [2, 6]. In horses, strangles infections that are complicated by increased pathogenesis should be treated with a penicillin [6].

Rarely have cases of human infection with *S. equi* subsp. *equi* been reported throughout the world, particullarly in the United States. Many cases of human infection with *S. equi* arise from the subsp. *zooepidemicus* [1, 7–10]. Bacterial meningitis due to *S. equi* appears to be one of the most common presentations of this infection, and these individuals often have a concomitant bacteremia [1]. Human infection with *S. equi* subsp. *equi* is frequently acute and has increased potential for high mortality, especially when individuals are immunocompromised, have a neurologic susceptibility, or cardiovascular disease [3, 4, 12–15]. A 13 year old who developed meningitis following exposure to *S. equi* in a sick pony was reported in 2020 [13]. This young boy also had a history of systemic lupus erythematosus, rendering him slightly immunocompromised and highlighting the correlation between *S. equi* infection and immunocompetence status. His infection was complicated by a subdural empyema. He made a full recovery with no long-term neurological deficits. In 2022, a 12 year old girl in Poland developed a retropharyngeal abscess colonized by *S. equi* subsp. *equi*, which was the first reported case of a *S. equi* infection of this sort [12]. A young boy in Canada developed meningitis following a *S. equi* subsp. *equi* infection in 2002 [14]. He lived on a horse farm with his parents and had a history of cranial surgery and hydrocephalus; thus, he may have been more susceptible to future central nervous system infections. Unfortunately, this patient did endure long-term complications of his infection, including long-term ataxia and sensorineural deafness, for which he received a cochlear implant [14]. Meningitis due to *S. equi* subsp. *equi* infection was also reported in a 75-year-old Romanian woman who had a cardiovascular history of cardiomyopathy and heart failure [15]. She had visited her neighbor’s horse farms within the two weeks prior to her illness. Lastly, a case was reported out of Belgium in 2021 reporting a meningoencephalitis infection with *S. equi* subsp. *equi*, eventually resulting in a significant central nervous system infection and a dural arteriovenous fistula [4]. This 69-year-old man had recently returned from a vacation where he rode and was exposed to horses. He initially recovered from the primary illness, but he returned to the hospital five months later with numerous cerebral hemorrhages. He did have a neurosurgical skull reconstruction following a fracture five years prior to this event, which may have predisposed him to neurological complications. However, he did survive with almost completely resolved neurological symptoms [4].

To the best of the authors’ knowledge, these five cases make up the breadth of reported *S. equi* subsp. *equi* infections worldwide. However, only one case of horse to human transmission of *S. equi* subsp. *equi* has been reported in the United States in the last 40 years, based on a literature review at the time of submission [5]. The current case would bring this number to two. It is important for both clinicians and individuals in higher risk environments to recognize the potential for strangles to spread from an infected horse to a human, especially if the individual is immunocompromised. Additionally, some longer term deficits have been noted in previous cases, particularly neurological deficits following a meningitis infection [1, 13]. Reported sequelae of *S. equi* infection include ataxia, deafness, amnesia, hemiparesis, or blindness [1, 4, 13]. These highlight the necessity of identifying a *S. equi* infection, including the value in gathering a zoonotic history from patients living in ranching or farming communities, individuals who regularly work with animals, or immunocompromised individuals presenting with signs of meningitis or bacteremia.

In our report, it is likely that the pathogen originally infected the patient as a sub-clinical pneumonia following her close contact with a horse, presumptively suffering from strangles six days prior to her emergency department visit. Whether the colonoscopy procedure contributed to the pathogen seeding into her blood stream or not is uncertain, but the patient was later bacteremic. The incidence of bacteremia following colonoscopy varies from 0 to 25%, with a mean frequency of 4.4% [16]. In reviewing the literature for this case, there does not appear to be data to suggest *S. equi* subsp. *equi* naturally occurs in the human gastrointestinal tract. Nonetheless, her initial presentation was consistent with early sepsis. Had it gone untreated, it could have become fatal. In this case, the patient presented with an early pneumonia that was not evident on chest radiograph. It appears in the literature that most cases have evolving symptomology and progression of the infection. This patient certainly had features indicating illness, however the early presentation and treatment may have been key in preventing further hematogenous spread. The patient was treated with piperacillin-tazobactam prior to the identification of *S. equi* in the blood culture, and this proved effective in treating the bacteremia. Per the patient’s blood counts and later blood culture, it appears that penicillin is the proper treatment for *S. equi* infections in both horses and humans [6].

This report discusses an atypical presentation of *S. equi* subsp. *equi* infection in an otherwise healthy individual, manifesting as early sepsis, pneumonia, and bacteremia. The patient likely developed this infection following direct contact exposure to her horse who had died from presumed strangles a few days prior to her symptom onset. The patient was successfully treated with penicillin and fully recovered from her bacteremia and pneumonia. This case highlights the importance of investigating potential exposures to *S. equi* in rural areas, areas where farming and ranching are prevalent, particularly among individuals working with horses. It is especially important to acknowledge high risk populations such as immunocompromised individuals with signs and symptoms of meningitis or bacteremia. Without medical intervention, infection with *S. equi* can cause significant mortality and morbidity for the individual [1, 4, 13]. In the most severe forms, *S. equi* infections are concerning for progression to meningitis, bacteremia, or death. Rapid identification of susceptible populations and possible exposures among humans is crucial to successfully treating a *S. equi* infection.

## Data Availability

Not applicable.
